# Interplay between Matrix Metalloproteinase-9, Matrix Metalloproteinase-2, and Interleukins in Multiple Sclerosis Patients

**DOI:** 10.1155/2016/3672353

**Published:** 2016-07-31

**Authors:** Alessandro Trentini, Massimiliano Castellazzi, Carlo Cervellati, Maria Cristina Manfrinato, Carmine Tamborino, Stefania Hanau, Carlo Alberto Volta, Eleonora Baldi, Vladimir Kostic, Jelena Drulovic, Enrico Granieri, Franco Dallocchio, Tiziana Bellini, Irena Dujmovic, Enrico Fainardi

**Affiliations:** ^1^Section of Medical Biochemistry, Molecular Biology and Genetics, Department of Biomedical and Specialist Surgical Sciences, University of Ferrara, 44121 Ferrara, Italy; ^2^Section of Neurology, Department of Biomedical and Specialist Surgical Sciences, University of Ferrara, 44121 Ferrara, Italy; ^3^Section of Orthopedics, Obstetrics and Gynecology and Anesthesia and Resuscitation, Department of Morphology, Surgery and Experimental Medicine, University of Ferrara, 44121 Ferrara, Italy; ^4^Neurology Unit, Department of Neurosciences and Rehabilitation, Azienda Ospedaliera-Universitaria, Arcispedale S. Anna, 44124 Ferrara, Italy; ^5^Neurology Clinic, Clinical Centre of Serbia, School of Medicine, University of Belgrade, Dr. Subotica 6, 11000 Belgrade, Serbia; ^6^Neuroradiology Unit, Department of Neurosciences and Rehabilitation, Azienda Ospedaliera-Universitaria, Arcispedale S. Anna, 44124 Ferrara, Italy

## Abstract

Matrix Metalloproteases (MMPs) and cytokines have been involved in the pathogenesis of multiple sclerosis (MS). However, no studies have still explored the possible associations between the two families of molecules. The present study aimed to evaluate the contribution of active MMP-9, active MMP-2, interleukin- (IL-) 17, IL-18, IL-23, and monocyte chemotactic proteins-3 to the pathogenesis of MS and the possible interconnections between MMPs and cytokines. The proteins were determined in the serum and cerebrospinal fluid (CSF) of 89 MS patients and 92 other neurological disorders (OND) controls. Serum active MMP-9 was increased in MS patients and OND controls compared to healthy subjects (*p* < 0.001 and *p* < 0.01, resp.), whereas active MMP-2 and ILs did not change. CSF MMP-9, but not MMP-2 or ILs, was selectively elevated in MS compared to OND (*p* < 0.01). Regarding the MMPs and cytokines intercorrelations, we found a significant association between CSF active MMP-2 and IL-18 (*r* = 0.3, *p* < 0.05), while MMP-9 did not show any associations with the cytokines examined. Collectively, our results suggest that active MMP-9, but not ILs, might be a surrogate marker for MS. In addition, interleukins and MMPs might synergistically cooperate in MS, indicating them as potential partners in the disease process.

## 1. Introduction

Multiple sclerosis (MS) is a disease of the central nervous system (CNS) of supposed autoimmune origin, characterized by inflammation, demyelination, and neurodegeneration [1]. Although the pathological features of the disease are heterogeneous, a common event is thought to be the reactivation within the CNS of infiltrating myelin-specific T cells which, in turn, trigger the recruitment of innate immunity cells mediating demyelination and axonal loss [2]. The perivascular transmigration and accumulation of inflammatory cells within the CNS are mainly mediated by two events: the production of leukocyte-attracting chemokines and the blood-brain barrier (BBB) breakdown [3]. The production of chemokines may be important for the regulation of the inflammatory cells influx to sites of tissue damage. Within the chemokine family, particularly studied members in the course of MS are the monocyte chemotactic proteins (MCPs), with MCP-1 and MCP-2 being selectively expressed at high levels in active lesions, while MCP-3 was mostly observed in the extracellular matrix surrounding the vascular elements [4]. In addition to the establishment of a chemokine gradient, the BBB has to be disrupted in order for the leukocytes to infiltrate within the CNS [5]. This event is mediated by the action of matrix metalloproteinases (MMPs), a family of Zn^2+^-dependent and Ca^2+^-requiring endopeptidases involved in the modeling of the extracellular matrix in both physiological and pathological conditions. Among all MMPs, MMP-9 and MMP-2 have been extensively studied in MS given their ability to degrade the components of the basal lamina and to mediate BBB damage [6–8].

Notably, growing experimental evidence suggests the involvement of MMP-9 in the pathogenesis of MS, where its circulating levels in serum and cerebrospinal fluid (CSF) were found to be upregulated in MS patients compared with noninflammatory neurological disorders (NIND) and healthy controls [9–13]. On the contrary, the implication of MMP-2 in the pathogenesis of MS is more controversial, since this enzyme had demonstrated both protective [7] and detrimental actions [14]. Besides MMPs, inflammatory cytokines, in particular the interleukins belonging to the Th17 axis, IL-23 and IL-17, might also play a role in MS [15].

IL-23, a member of the IL-12 cytokine family, is a heterodimeric protein with the ability to support the polarization and expansion of T cells toward a Th17 phenotype [16, 17]. Its involvement in the pathogenesis of MS has been suggested by evidence from the animal model of the disease, the experimental autoimmune encephalomyelitis (EAE). Indeed, this cytokine has proven to be essential for the development of EAE [18] and the transfer of Th17 cells, polarized and expanded by IL-23, was able to induce the disease in animals [19]. Th17 cells are strictly connected to the pathogenesis of MS through, but not limited to, the production of several proinflammatory cytokines including IL-17 (A and F), which has been found upregulated in chronic lesions of MS patients [20] and in the serum of Interferon-*β* (IFN-*β*) nonresponding patients [21]. In addition to the abovementioned factors, IL-18, another cytokine important in Th1 response in the course of MS [22], has been found increased in serum and CSF of MS patients compared to noninflammatory controls, with the levels of the molecule being higher in those with MRI gadolinium enhancing lesions [23]. Nonetheless, several animal and* in vitro* evidence connected both MMPs to IL-18 [24] and to the IL-17/IL-23 axis [25], demonstrating a general stimulating effect on the enzymes production, whereas other reports suggested a regulation of MMP-2 on MCP-3 activity [26] showing an anti-inflammatory effect [27]. However, to the best of our knowledge none of the previous studies evaluated the possible interrelationships between the active forms of MMP-9 and MMP-2 and the most common cytokines involved in MS pathogenesis. Therefore, in the present study our aim was to measure the levels of active MMP-9 and MMP-2, IL-17, IL-18, IL-23, and MCP-3 in the serum and CSF of MS patients and controls in order to investigate the contribution of these molecules to MS pathogenesis. Moreover, we aimed to explore possible interrelationships between cytokines, MMPs, and clinical variables.

## 2. Material and Methods

### 2.1. Patients Selection

For this study, we recruited 89 consecutive patients affected by definite relapsing-remitting MS (RRMS), according to McDonald criteria [28], who presented at the Neurology Clinic of the University of Belgrade. Evidence of a relapse at admission was considered clinical disease activity [29]. The data were available for a total of 40 patients out of 89. Accordingly, 32 patients were clinically active, whereas 8 patients were clinically stable. Patient disease severity was measured by Kurtzke's Expanded Disability Status Scale (EDSS) [30]. Disease duration was scored and expressed in years. At the time of sample collection, none of the patients had fever or other signs of acute infection, nor had they been receiving any disease-modifying therapies (DMTs) during the 6 months before the study. A total of 92 controls with other neurological disorders (OND) were also included in the study (Table 1). OND patients were free of immunosuppressant drugs, including steroids, at the time of sample collection. In addition, a total of 40 age- and sex-matched healthy controls (HC) were used. Informed consent was given by all patients before inclusion in the study and the study design was approved by the Ethics Committee of the School of Medicine, University of Belgrade.

### 2.2. CSF and Serum Sampling

Cerebrospinal fluid and serum samples were collected under sterile conditions and stored in aliquots at −80°C until assay. “Cell-free” CSF samples were obtained after centrifugation at room temperature of specimens taken by lumbar puncture performed for diagnosis purposes. Serum samples were derived from centrifugation of blood specimens withdrawn by puncture of an anterocubital vein at the same time of CSF extraction. Paired CSF and serum samples from RRMS and OND patients were stored and measured under exactly the same conditions. For the healthy controls, only the serum was available.

### 2.3. Assay of Interleukins in Serum and CSF

IL-17A, IL-23, and MCP-3 levels were simultaneously measured in sera and CSF of patients, twofold diluted with dilution buffer or undiluted, respectively, by a multiplex sandwich enzyme-linked immunosorbent assay (ELISA) system based on chemiluminescence detection (Aushon SearchLight chemiluminescent assay kits, Tema Ricerca, Italy) according to the manufacturer's recommendations. All samples were analyzed in duplicate. The interleukin levels are reported as pg/mL. The lower concentration of each standard curve was 0.78 pg/mL for IL-17A, 19.5 pg/mL for IL-23, and 0.78 pg/mL for MCP-3.

IL-18 was measured in CSF and serum samples, twofold diluted, with commercially available ELISA (Boster Immunoleader cod. EK0864). Samples were assayed in duplicate. A standard curve was generated in each plate and the lower standard concentration was 15.6 pg/mL.

### 2.4. Assay of Active MMP-9 in Serum and CSF

Serum and CSF levels of circulating active MMP-9 were determined using a commercially available activity assay system (Human Active MMP-9 Fluorokine E Kit, R&D systems; Cat. Number F9M00) following the manufacturer's instructions. All the reagents were included in the kit. For the determinations, a standard curve in the range of 16–0.125 ng/mL was used; serum and CSF samples were diluted 100 times and 2 times, respectively, with the calibrator diluent (RD5-24) included in the kit. According to the manufacturer's data, the minimum detectable dose was 0.005 ng/mL and the range of intra-assay and interassay coefficient of variation (CV) was 3.9–4.8% and 8.0–9.3%, respectively.

### 2.5. Assay of Active MMP-2 in Serum and CSF

Serum and CSF levels of circulating active MMP-2 were determined using a commercially available activity assay system (MMP-2, Biotrak Activity Assay System, GE Healthcare; Cat. Number RPN2631) following the manufacturer's instructions. All the reagents were included in the kit. For the determinations, a standard curve in the range of 4–0.125 ng/mL was used; serum and CSF samples were diluted 25 times and 2 times, respectively, with the assay buffer included in the kit. According to the manufacturer's data, the sensitivity was 0.190 ng/mL and the range of intra-assay and interassay coefficient of variation (CV) was 4.4–7.0% and 16.9–18.5%, respectively.

### 2.6. Statistical Analysis

Normality of distribution was checked by Shapiro-Wilk test. Since the variables were not normally distributed, group comparisons were performed using Kruskal-Wallis followed by Mann-Whitney* U* tests, with Bonferroni correction for multiple comparisons. Bivariate correlations were performed by Spearman's rank test and frequency distributions were examined using the Chi-square test. To assess the association between abnormal MMPs and ILs values measured in serum or CSF and the MS pathology, a binary logistic regression analysis was performed. A value of *p* < 0.05 was considered statistically significant.

## 3. Results

### 3.1. Active MMP-9 and MMP-2 in Serum and CSF of MS Patients and Controls

**Figure 1:**
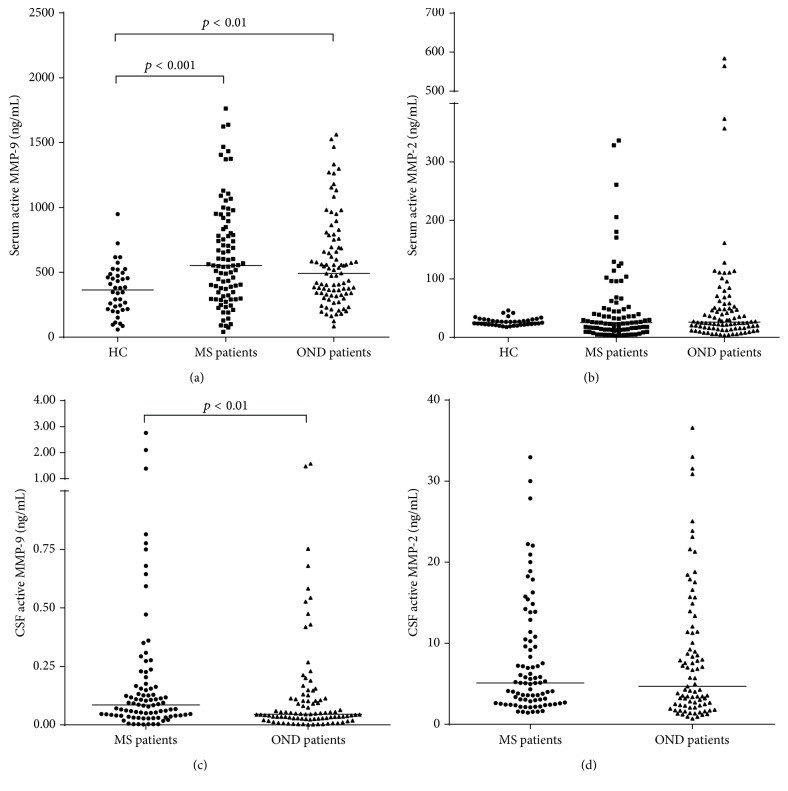
Median of serum total active MMP-9 and MMP-2, in RRMS patients, OND controls, and healthy donors and CSF active MMP-9 and MMP-2 in RRMS patients and OND controls. Serum levels of total active MMP-9 were statistically different among the groups (Kruskal-Wallis; H(2) = 15.45, *p* < 0.0001) and CSF active MMP-9 levels were elevated in RRMS patients compared to OND patients. (a) Serum concentrations of total active MMP-9 were not different among RRMS (median (IQR): 552 (318–841) ng/mL) and OND (492 (330–737) ng/mL) patients; whereas they were higher (Mann Whitney; *p* < 0.001 and *p* < 0.01) in RRMS and OND patients when compared to HC (363 (216–482) ng/mL). (b) Serum levels of active MMP-2 were not different between RRMS patients (25.2 (13.1–48.1) ng/mL), OND patients (26.2 (15.8–52.5) ng/mL), and HC (25.8 (21.8–30.7) ng/mL). (c) CSF amounts of active MMP-9 were more increased in RRMS (0.084 (0.040–0.165) ng/mL) than in OND (0.046 (0.027–0.113) ng/mL) patients (Mann Whitney; *p* = 0.009). (d) CSF levels of active MMP-2 were not different between RRMS (5.1 (2.7–10.4) ng/mL) and OND (4.7 (2.3–11.4) ng/mL) controls. IQR: interquartile range; HC: healthy controls; MMP: matrix metalloproteinase; RRMS: relapsing-remitting MS; OND: other neurologic disorders; CSF: cerebrospinal fluid.

Active MMP-9 and MMP-2 were detectable in 100% of serum samples and in 100% and in 93% (85 OND and 84 MS) of CSF samples for MMP-9 and MMP-2, respectively. As reported in Figure 1(a), the levels of active MMP-9 were different among the groups. In particular, we found a higher concentration of active MMP-9 in the serum of both MS patients and OND controls compared to healthy subjects (*p* < 0.001 and *p* < 0.01, resp.). On the contrary, active MMP-2 serum levels were similar in MS, OND, and healthy subjects (Figure 1(b), Kruskal-Wallis *H*(2) = 1.009, *p* = 0.604). Then, we compared the amounts of both active gelatinases measured in the CSF of MS patients and OND controls. As depicted in Figure 1(c), MS patients showed almost a doubled concentration of active MMP-9 compared to OND controls (*p* = 0.009), whereas the levels of active MMP-2 did not differ (Figure 1(d), median (interquartile range): 4.7 (2.3–11.4) and 5.1 (2.7–10.4) for OND and MS patients, resp.; *p* = 0.713). When patients were grouped according to clinical disease activity, there were no statistical differences between MS patients with and without clinical evidence of disease activity, for both serum and CSF active MMP-9 and MMP-2 (data not shown).

### 3.2. Interleukin Levels in Serum and CSF of MS Patients and Controls

**Figure 2:**
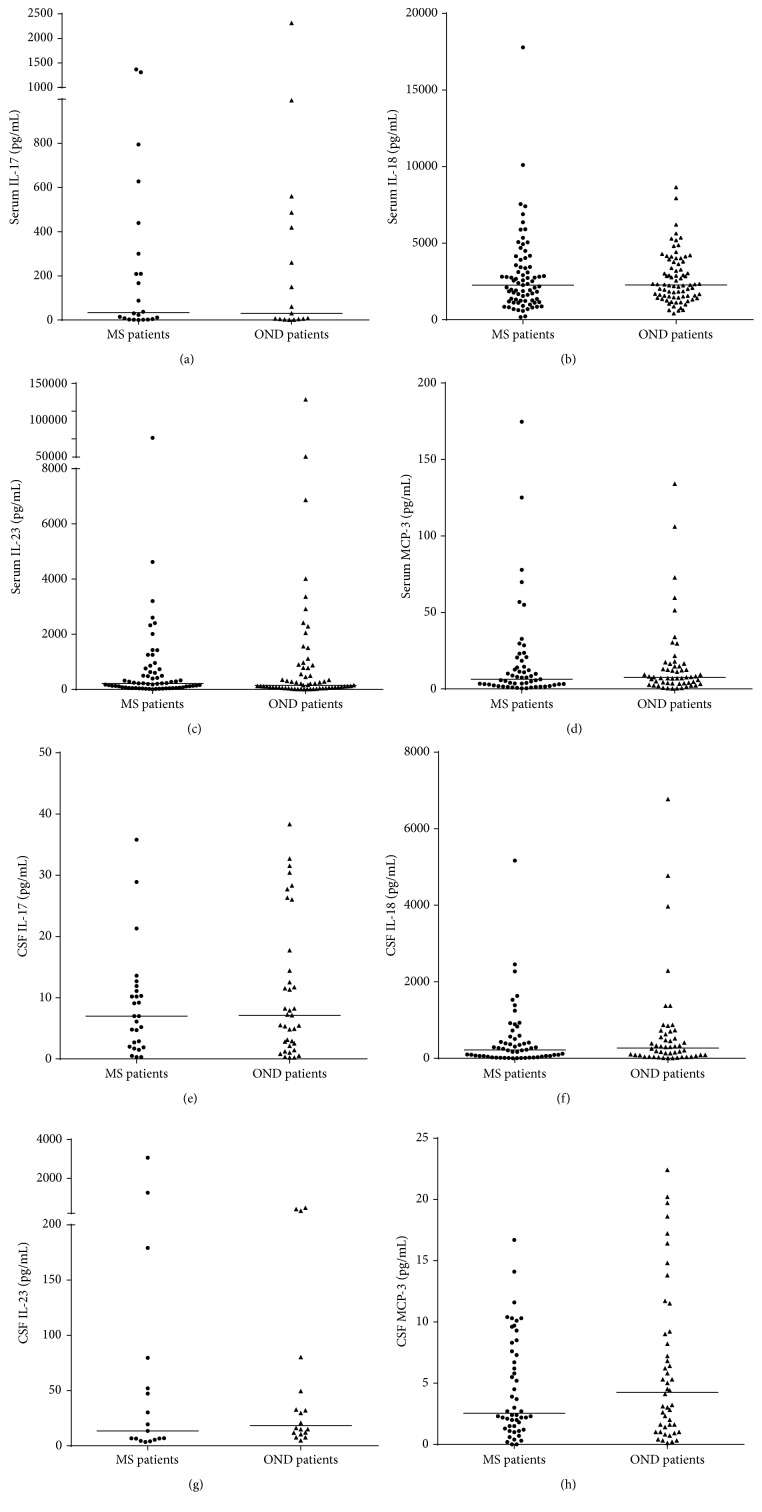
Median of serum and CSF IL-17, IL-18, IL-23, and MCP-3 concentrations in RRMS patients and OND controls. None of the examined cytokines/chemokines was different between RRMS patients and OND patients in either the serum or CSF. (a) Serum levels of IL-17 in RRMS (median (IQR): 33.7 (4.2–335.0) pg/mL) and OND (44.8 (4.1–468.0) pg/mL) patients. (b) Serum levels of IL-18 in RRMS (2259 (1232–3505) pg/mL) and OND (2266 (1509–3766) pg/mL) patients. (c) Serum levels of IL-23 in RRMS (212.3 (64.9–625.3) pg/mL) and OND (148.9 (54.6–774.9) pg/mL) patients. (d) Serum levels of MCP-3 in RRMS (6.3 (2.6–18.4) pg/mL) and OND (7.5 (3.5–14.7) pg/mL) patients. (e) CSF levels of IL-17 in RRMS (7.0 (2.0–11.1) pg/mL) and OND (7.1 (2.3–16.1) pg/mL) patients. (f) CSF levels of IL-18 in RRMS (218 (49–551) pg/mL) and OND (269 (71–644) pg/mL) patients. (g) CSF levels of IL-23 in RRMS (13.4 (5.9–65.9) pg/mL) and OND (18.2 (11.4–57.1) pg/mL) patients. (h) CSF levels of MCP-3 in RRMS (2.6 (1.5–7.8) pg/mL) and OND (4.3 (1.3–9.1) pg/mL) patients. IQR: interquartile range; CSF: cerebrospinal fluid; IL: interleukin; MCP: Monocyte Chemoattractant Protein; RRMS: relapsing-remitting MS; OND: other neurological disorders.

The levels of IL-17, IL-18, IL-23, and MCP-3 in the serum of OND and MS patients were detectable in 21% of samples for IL-17 (16 OND and 22 MS), 86% for IL-18 (79 OND and 77 MS), 71% for IL-23 (65 OND and 64 MS), and 61% for MCP-3 (55 OND and 55 MS). In the CSF, the values were detectable in 35% of samples for IL-17 (35 OND and 27 MS), 59% for IL-18 (50 OND and 56 MS), 19% for IL-23 (18 OND and 17 MS), and 53% for MCP-3 (46 OND and 50 MS). As reported in Figures 2(a)–2(d), we did not find any significant difference in the serum concentration of the measured cytokines. The same result was observed in the CSF, where the levels of the cytokines were not different between the OND controls and the MS patients (Figures 2(e)–2(h)). When patients were grouped according to clinical disease activity, we did not find any statistical differences between MS patients with and without clinical evidence of disease activity, for both serum and CSF IL-17, IL-18, IL-23, and MCP-3 levels (data not shown).

### 3.3. Correlations between Interleukin Levels and Active MMP-9 and MMP-2 in Serum and CSF of MS Patients and with Clinical Outcomes

We evaluated possible correlations between the levels of MMPs and interleukins measured in the serum of MS patients. As reported in Table 2, we observed significant positive correlations between MCP-3 and IL-17, between MCP-3 and IL-23, and between IL-17 and IL-23. Of note, we did not find any relation between the active forms of MMPs and the interleukins, although there was a tendency toward a significant negative correlation between serum active MMP-9 and IL-18 (*p* = 0.076).

Then, we evaluated the correlations between active MMPs and interleukins measured in the CSF of patients. The results are summarized in Table 3. Notably, we found a positive correlation between IL-18 and active MMP-2 and MCP-3 and IL-17 and between IL-18 and IL-23.

There were no significant correlations between disease severity scored by EDSS, disease duration, and serum and CSF levels of the measured proteins.

### 3.4. Evaluation of Abnormal MMPs and Interleukin Levels in Serum and CSF of MS Patients and Controls

Based on the median values of the active MMPs and cytokines measured in the serum or CSF from the whole population, we determined in all subjects whether the active MMP-9 or cytokines were increased or active MMP-2 was decreased. These values were considered abnormal. In addition, the cytokine abnormal values were merged in one category (Table 4,* combined interleukins*), including subjects with at least one abnormal value of IL-17, IL-18, IL-23, or MCP-3.

As shown in Table 4, we did not find any difference in the frequency of abnormal levels of either interleukins or MMPs in serum. The same result was observed when we analyzed the frequency of patients with abnormal CSF levels of the considered proteins, with the exception of the active MMP-9. Indeed, there was a higher proportion of MS patients with abnormally increased levels of the enzyme compared to OND controls (Pearson Chi-square (1): 9.335, *p* = 0.002). Then, we searched for possible association of abnormal levels of MMPs and interleukins with MS by employing a binary logistic regression analysis, considering the diagnosis (MS or OND) as the outcome variable and entering the abnormal levels of MMPs or cytokines alone or in combination (*combined interleukins*) as predictors. From this analysis, no association emerged between serum active MMP-9, active MMP-2, or the cytokines and MS pathology. On the contrary when we analyzed the proteins measured in the CSF, we found that only the abnormal values of active MMP-9 were associated with an increased likelihood of being affected by MS (Odds Ratio: 2.52, 95% Confidence Interval: 1.39–4.59, *p* = 0.002). Of note, the inclusion of the other covariates did not improve the reliability of the model (data not shown).

Finally, we compared the levels of serum or CSF active MMP-9 and MMP-2 measured in MS patients, grouped according to the abnormal level of cytokines alone or in combination. This analysis did not show any difference in the concentrations of the two MMPs between the patients with normal or high values of ILs either in serum or in CSF.

## 4. Discussion

There is evidence connecting both MMPs and Th17/Th1-related cytokines to the pathogenesis of MS. Indeed, both families of proteins have been advocated as markers of disease activity [31–33], for therapeutic response [34] and as active players in the MS disease course [15, 35]. In particular, MMPs are involved in both BBB disruption and formation of MS lesions [36], whereas cytokines and chemokines may play important roles in the recruitment of leukocytes into the CNS [4] and in the initiation of the autoimmune tissue inflammation [15]. Nevertheless, MMPs and cytokines/chemokines may also cooperate in the opening of the BBB, a key event that can further support the leukocyte migration within the CNS [37]. Notwithstanding the accumulating evidence that might suggest a possible interplay between MMPs and cytokines, there is still a lack of clinical studies exploring possible associations between the mentioned molecules in the serum and CSF of MS patients.

In light of the above considerations, we set out the present study with the aim to evaluate the contribution of MMPs, namely, active MMP-9 and MMP-2, and the cytokines/chemokines IL-17, IL-18, IL-23, and MCP-3 to the pathogenesis of MS. More importantly, for the first time we evaluated the possible intercorrelations involving these two classes of molecules. In agreement with previous studies [31, 38], we found that serum active MMP-9 was higher in patients with MS and OND compared to healthy controls, whereas the CSF active MMP-9 was selectively elevated in MS patients. On the contrary, our finding of the lack of association between serum and CSF levels of active MMP-2 and MS disagrees with previous observations [32]. In our view, divergences in patient selection or genetic and environmental factors [39] might partially explain these conflicting results.

The lack of difference we found in the serum and CSF cytokine concentrations also appears in contradiction with previous reports showing higher serum levels of IL-23 and IL-18 in MS patients compared to healthy controls [23, 34, 40]. Moreover, other studies showed increased [40] but also unchanged [34] levels of IL-17 in the serum or PBMC [41] of MS patients compared to controls. This apparent discrepancy might be due to a different selection in the control group, since, at variance of ours, most of the studies compared MS patients with heathy donors, and to the low detectable rate of cytokines in both serum and CSF. Of note, to the best of our knowledge, there are no data in literature about MCP-3 circulating levels.

The observed strong correlations between IL-17, IL-23, and MCP-3 in the serum and between MCP-3, IL-17, IL-18, and IL-23 in the CSF of MS patients suggest that, though not massive, the MS pathology might be characterized by a general overproduction of cytokines and chemokines. However, if the overproduction occurs it remains within the normal values measured in OND controls, since we did not find any difference in the proportion of abnormal cytokine levels between the two groups. Collectively, these results suggest that, at least in our cohort, cytokines might represent poor surrogate markers of the disease.

On the contrary, active MMP-9, which was selectively elevated in the CSF of MS patients, could be considered as appropriate indicator of ongoing inflammation in MS. Consistently, we found a higher proportion of MS patients with abnormal CSF levels of active MMP-9 compared to OND patients, with an increased likelihood of being affected by MS (Odds Ratio: 2.52). However, the missed correlation between active MMP-9 and cytokines in either the serum or CSF (although likely due to the low detection rate of cytokines) suggests that the activation cascade of the enzyme might not act in concert with these soluble proinflammatory factors in the course of MS.

On the other hand, the significant positive correlation between active MMP-2 and IL-18 suggests that this proinflammatory cytokine might be able to modulate the activation cascade of MMP-2. In line with this hypothesis, a recent* in vitro* study on neuron-like cells reported an upregulation of MMP-14, the physiological activator of MMP-2, upon treatment with increasing amounts of IL-18 [42]. However, this finding seems in contradiction with the supposed major role of active MMP-2 in the resolution phase of the disease, where its levels were lower in patients with MRI evidence of disease activity [32] indicating a possible anti-inflammatory action [26]. Of note, we did not find any difference in both MMPs and cytokine levels between clinically active and inactive MS patients, suggesting that these molecules do not seem correlated to clinical exacerbations. However, it is well known that MRI is superior on clinical examination in measuring MS disease activity [43], and thus we cannot exclude that the real contribution of these proteins to the disease pathogenesis has been underestimated. Indeed, in previous reports [32, 38] we observed differences in active MMPs only when patients were categorized in active or inactive disease based on MRI findings.

This study was not without its limitations. First, the small sample size may have weakened the consistency of our data, making it difficult to draw any definitive conclusion about the possible use of the analyzed molecules as reliable biomarkers of the disease. Second, the design of the study was cross-sectional, thereby precluding our ability to establish any real cause/effect relationship between the cytokines and MMPs. A longitudinal approach could be more suitable. Third, the lack of complete data on the clinical activity of the disease may have mined the ability to detect real differences between clinically active and stable MS patients. However, in previous studies we did not find significant differences when patients were grouped according to clinical evidence of disease activity [31, 32]. Fourth, the lack of MRI examinations in our study could have affected our findings. Finally, the number of patients and controls with detectable levels of cytokines was low, limiting the reliability of our results. Consequently, a replication of the data in larger cohorts of MS patients and controls is warranted.

In conclusion, although with limitations, our study confirms that active MMP-9 could be a potential surrogate marker for monitoring MS disease, whereas cytokines and chemokines seem not able to discriminate between MS patients and controls. Nonetheless, our results also highlighted that MMPs and cytokines might synergistically cooperate in MS, indicating them as potential partners in the disease processes. Further studies in a larger number of patients are needed to verify the effective nature and role of this cooperation in the modulation of the inflammatory responses operating in MS.

## Figures and Tables

**Table 1:** Demographic and clinical characteristics of healthy controls and OND and RRMS patients.

	Healthy controls (*n* = 40)	OND (*n* = 92)	MS (*n* = 89)
Age	37.0 ± 7.5; 35.5 (30.3–44.0)	42.4 ± 13.7; 41.5 (33.0–49.0)	39.2 ± 10.9; 37.0 (30.5–48.0)
Sex: female/male	24/16	57/35	53/36
Disease duration (yrs)	—	—	5.9 ± 7.1; 3 (1–7.7)
EDSS	—	—	3.8 ± 1.9; 3.5 (2.5–4.4)
Clinically active MS: *n*/total (%)	—	—	32/40 (80%)
Clinically stable MS: *n*/total (%)	—	—	8/40 (20%)

EDSS: expanded disability status scale; MS: multiple sclerosis; RRMS: relapsing-remitting multiple sclerosis; OND: other neurological disorders.

**Table 2:** Correlation matrix of active MMP-9, active MMP-2, and interleukins measured in the serum of MS patients.

Variables	(1)	(2)	(3)	(4)	(5)	(6)
(1) Active MMP-9	—					
(2) Active MMP-2	−0.166 (89)	—				
(3) IL-17	−0.207 (22)	0.290 (22)	—			
(4) IL-18	−0.204 (77)	0.132 (77)	0.387 (22)	—		
(5) IL-23	−0.196 (64)	−0.017 (64)	0.466 (22)^*∗*^	−0.138 (63)	—	
(6) MCP-3	−0.128 (55)	0.028 (55)	0.922 (21)^*∗∗*^	0.212 (55)	0.468 (48)^*∗∗*^	—

Values in brackets represent the degrees of freedom. ^*∗*^ *p* < 0.05; ^*∗∗*^ *p* < 0.01.

**Table 3:** Correlation matrix of active MMP-9, active MMP-2, and interleukins measured in the CSF of MS patients.

	(1)	(2)	(3)	(4)	(5)	(6)
(1) Active MMP-9	—					
(2) Active MMP-2	0.244 (84)	—				
(3) IL-17	−0.306 (27)	−0.129 (25)	—			
(4) IL-18	−0.076 (56)	0.300 (53)^*∗*^	0.437 (19)	—		
(5) IL-23	0.075 (17)	−0.344 (16)	0.450 (7)	0.248 (10)	—	
(6) MCP-3	−0.127 (50)	0.237 (47)	0.485 (20)^*∗*^	0.454 (33)^*∗∗*^	0.575 (13)^*∗*^	—

Values in brackets represent the degrees of freedom. ^*∗*^ *p* < 0.05; ^*∗∗*^ *p* < 0.01.

**Table 4:** Percentage of MS patients and OND controls with abnormal serum and CSF values.

	OND (%)	MS (%)
*Serum*		
High active MMP-9	46.7	53.9
Low active MMP-2	53.3	49.4
High IL-17	8.7	12.4
High IL-18	43.5	42.7
High IL-23	32.6	38.2
High MCP-3	30.4	30.3
*Combined interleukins*	*63.0*	*69.7*

*CSF*		
High active MMP-9	40.2	62.9^*∗∗*^
Low active MMP-2	51.8	47.6
High IL-17	20.7	13.5
High IL-18	28.3	30.3
High IL-23	9.8	9.0
High MCP-3	28.3	24.7
*Combined interleukins*	*45.7*	*44.9*

The cut-off values used for the determination of the frequency of abnormal values were as follows.

Serum: active MMP-9, 534 ng/mL; active MMP-2, 25.2 ng/mL; IL-17, 33.6 pg/mL; IL-18, 2262 pg/mL; IL-23, 181 pg/mL; MCP-3, 7.2 pg/mL.

CSF: active MMP-9, 0.055 ng/mL; active MMP-2, 5.06 ng/mL; IL-17, 7 pg/mL; IL-18, 237 pg/mL; IL-23, 15.9 pg/mL; MCP-3, 3 pg/mL.

^*∗∗*^ *p* < 0.01.

## References

[1] Compston A., Coles A. (2008). Multiple sclerosis. *The Lancet*.

[2] Goverman J. (2009). Autoimmune T cell responses in the central nervous system. *Nature Reviews Immunology*.

[3] Wilson E. H., Weninger W., Hunter C. A. (2010). Trafficking of immune cells in the central nervous system. *The Journal of Clinical Investigation*.

[4] McManus C., Berman J. W., Brett F. M., Staunton H., Farrell M., Brosnan C. F. (1998). MCP-1, MCP-2 and MCP-3 expression in multiple sclerosis lesions: an immunohistochemical and in situ hybridization study. *Journal of Neuroimmunology*.

[5] Dos Passos G. R., Sato D. K., Becker J., Fujihara K. (2016). Th17 cells pathways in multiple sclerosis and neuromyelitis optica spectrum disorders: pathophysiological and therapeutic implications. *Mediators of Inflammation*.

[6] Minagar A., Alexander J. S. (2003). Blood-brain barrier disruption in multiple sclerosis. *Multiple Sclerosis*.

[7] Yong V. W. (2005). Metalloproteinases: mediators of pathology and regeneration in the CNS. *Nature Reviews Neuroscience*.

[8] Lau L. W., Cua R., Keough M. B., Haylock-Jacobs S., Yong V. W. (2013). Pathophysiology of the brain extracellular matrix: a new target for remyelination. *Nature Reviews Neuroscience*.

[9] Leppert D., Ford J., Stabler G. (1998). Matrix metalloproteinase-9 (gelatinase B) is selectively elevated in CSF during relapses and stable phases of multiple sclerosis. *Brain*.

[10] Lee M. A., Palace J., Stabler G., Ford J., Gearing A., Miller K. (1999). Serum gelatinase B, TIMP-1 and TIMP-2 levels in multiple sclerosis. A longitudinal clinical and MRI study. *Brain*.

[11] Waubant E., Goodkin D. E., Gee L. (1999). Serum MMP-9 and TIMP-1 levels are related to MRI activity in relapsing multiple sclerosis. *Neurology*.

[12] Liuzzi G. M., Trojano M., Fanelli M. (2002). Intrathecal synthesis of matrix metalloproteinase-9 in patients with multiple sclerosis: implication for pathogenesis. *Multiple Sclerosis*.

[13] Benešová Y., Vašků A., Novotná H. (2009). Matrix metalloproteinase-9 and matrix metalloproteinase-2 as biomarkers of various courses in multiple sclerosis. *Multiple Sclerosis*.

[14] Yong V. W., Zabad R. K., Agrawal S., Goncalves DaSilva A., Metz L. M. (2007). Elevation of matrix metalloproteinases (MMPs) in multiple sclerosis and impact of immunomodulators. *Journal of the Neurological Sciences*.

[15] Amedei A., Prisco D., D'Elios M. M. (2012). Multiple sclerosis: the role of cytokines in pathogenesis and in therapies. *International Journal of Molecular Sciences*.

[16] Yang L., Anderson D. E., Baecher-Allan C. (2008). IL-21 and TGF-*β* are required for differentiation of human T_H_17 cells. *Nature*.

[17] Stritesky G. L., Yeh N., Kaplan M. H. (2008). IL-23 promotes maintenance but not commitment to the Th17 lineage. *Journal of Immunology*.

[18] Cua D. J., Sherlock J., Chen Y. (2003). Interleukin-23 rather than interleukin-12 is the critical cytokine for autoimmune inflammation of the brain. *Nature*.

[19] Langrish C. L., Chen Y., Blumenschein W. M. (2005). IL-23 drives a pathogenic T cell population that induces autoimmune inflammation. *Journal of Experimental Medicine*.

[20] Lock C., Hermans G., Pedotti R. (2002). Gene-microarray analysis of multiple sclerosis lesions yields new targets validated in autoimmune encephalomyelitis. *Nature Medicine*.

[21] Axtell R. C., De Jong B. A., Boniface K. (2010). T helper type 1 and 17 cells determine efficacy of interferon-Β in multiple sclerosis and experimental encephalomyelitis. *Nature Medicine*.

[22] Alboni S., Cervia D., Sugama S., Conti B. (2010). Interleukin 18 in the CNS. *Journal of Neuroinflammation*.

[23] Losy J., Niezgoda A. (2001). IL-18 in patients with multiple sclerosis. *Acta Neurologica Scandinavica*.

[24] Ishida Y., Migita K., Izumi Y. (2004). The role of IL-18 in the modulation of matrix metalloproteinases and migration of human natural killer (NK) cells. *FEBS Letters*.

[25] Song J., Wu C., Korpos E. (2015). Focal MMP-2 and MMP-9 activity at the blood-brain barrier promotes chemokine-induced leukocyte migration. *Cell Reports*.

[26] McQuibban G. A., Gong J.-H., Wong J. P., Wallace J. L., Clark-Lewis I., Overall C. M. (2002). Matrix metalloproteinase processing of monocyte chemoattractant proteins generates CC chemokine receptor antagonists with anti-inflammatory properties in vivo. *Blood*.

[27] Westermann D., Savvatis K., Lindner D. (2011). Reduced degradation of the chemokine MCP-3 by matrix metalloproteinase-2 exacerbates myocardial inflammation in experimental viral cardiomyopathy. *Circulation*.

[28] McDonald W. I., Compston A., Edan G. (2001). Recommended diagnostic criteria for multiple sclerosis: guidelines from the international panel on the diagnosis of multiple sclerosis. *Annals of Neurology*.

[29] Poser C. M., Paty D. W., Scheinberg L. (1983). New diagnostic criteria for multiple sclerosis: guidelines for research protocols. *Annals of Neurology*.

[30] Kurtzke J. F. (1983). Rating neurologic impairment in multiple sclerosis: an expanded disability status scale (EDSS). *Neurology*.

[31] Fainardi E., Castellazzi M., Bellini T. (2006). Cerebrospinal fluid and serum levels and intrathecal production of active matrix metalloproteinase-9 (MMP-9) as markers of disease activity in patients with multiple sclerosis. *Multiple Sclerosis*.

[32] Fainardi E., Castellazzi M., Tamborino C. (2009). Potential relevance of cerebrospinal fluid and serum levels and intrathecal synthesis of active matrix metalloproteinase-2 (MMP-2) as markers of disease remission in patients with multiple sclerosis. *Multiple Sclerosis*.

[33] Kallaur A. P., Oliveira S. R., Simão A. N. C. (2013). Cytokine profile in relapsing-remitting Multiple sclerosis patients and the association between progression and activity of the disease. *Molecular Medicine Reports*.

[34] Alexander J. S., Harris M. K., Wells S. R. (2010). Alterations in serum MMP-8, MMP-9, IL-12p40 and IL-23 in multiple sclerosis patients treated with interferon-*β*1b. *Multiple Sclerosis*.

[35] Opdenakker G., Van Damme J. (2011). Probing cytokines, chemokines and matrix metalloproteinases towards better immunotherapies of multiple sclerosis. *Cytokine and Growth Factor Reviews*.

[36] Romi F., Helgeland G., Gilhus N. E. (2012). Serum levels of matrix metalloproteinases: implications in clinical neurology. *European Neurology*.

[37] Larochelle C., Alvarez J. I., Prat A. (2011). How do immune cells overcome the blood-brain barrier in multiple sclerosis?. *FEBS Letters*.

[38] Trentini A., Manfrinato M. C., Castellazzi M. (2015). TIMP-1 resistant matrix metalloproteinase-9 is the predominant serum active isoform associated with MRI activity in patients with multiple sclerosis. *Multiple Sclerosis*.

[39] Gonçalves F. M., Martins-Oliveira A., Lacchini R. (2013). Matrix metalloproteinase (MMP)-2 gene polymorphisms affect circulating MMP-2 levels in patients with migraine with aura. *Gene*.

[40] Chen Y.-C., Chen S.-D., Miao L. (2012). Serum levels of interleukin (IL)-18, IL-23 and IL-17 in Chinese patients with multiple sclerosis. *Journal of Neuroimmunology*.

[41] Matusevicius D., Kivisäkk P., He B. (1999). Interleukin-17 mRNA expression in blood and CSF mononuclear cells is augmented in multiple sclerosis. *Multiple Sclerosis*.

[42] Sutinen E. M., Korolainen M. A., Häyrinen J. (2014). Interleukin-18 alters protein expressions of neurodegenerative diseases-linked proteins in human SH-SY5Y neuron-like cells. *Frontiers in Cellular Neuroscience*.

[43] Miller D. H., Barkhof F., Nauta J. J. P. (1993). Gadolinium enhancement increases the sensitivity of MRI in detecting disease activity in multiple sclerosis. *Brain*.

